# Exposure to 10 Hz Pulsed Magnetic Fields Do Not Induce Cellular Senescence in Human Fetal Lung Fibroblasts

**DOI:** 10.3389/fpubh.2021.761069

**Published:** 2021-11-11

**Authors:** Chuan Sun, Zheng Huang, Houbing Qin, Jing Zhang, Sanying Wang, Xiaogang Xu, Shibo Ying, Genxiang Mao

**Affiliations:** ^1^Zhejiang Provincial Key Lab of Geriatrics and Geriatrics Institute of Zhejiang Province, Department of Geriatrics, Zhejiang Hospital, Hangzhou, China; ^2^School of Stomatology, Hangzhou Normal University, Hangzhou, China; ^3^Department of Respiratory Medicine, Children's Hospital of Nanjing Medical University, Nanjing, China; ^4^Hangzhou Medical College, Hangzhou, China

**Keywords:** electromagnetic fields (EMFs), aging, DNA damage, pulsed electromagnetic field, cellular senescence, human fetal lung fibroblasts

## Abstract

Rapid population aging has led to a global burden of late-life diseases. As the largest risk factor for a multitude of age-related diseases, aging is not only the result of genotype but also closely related to external factors. With the rapid expansion in the usage of electromagnetic fields (EMFs), the effect of EMFs on aging has also attracted attention. Cells are the basic unit of organs and body tissues, and cellular senescence plays an important role in the aging process. The effect of EMFs on cellular senescence has been investigated in a few studies, but the information is limited, and the results are inconsistent; thus, further investigation is required. In this study, we investigated the effect of 10 Hz pulsed magnetic fields (MFs) on cellular senescence in a 2BS cell line, isolated from human fetal lung fibroblasts, and found that intermittent (1 d on/1 d off) exposure to 10 Hz pulsed MFs at 1.0 mT for 2 weeks induced DNA damage, but no other significant phenotype of cellular senescence in 2BS cells.

## Introduction

Nowadays, modern electromagnetic technology is developing rapidly, and humans are constantly exposed to various types of electromagnetic fields (EMFs) or magnetic fields (MFs) originating from different man-made sources. The possible adverse effects of EMFs on human health have attracted significant public attention (1, 2). Although non-ionizing EMFs do not have enough energy to directly ionize bio-macromolecules, many institutions, such as, the WHO, have issued warnings regarding their potential biological effects (3, 4). The International Agency for Research on Cancer (IARC) has categorized both radiofrequency and extremely low-frequency EMFs as possible carcinogens to humans and has warned the public about the potential risks (5, 6).

As the largest risk factor for a multitude of age-related diseases, aging is becoming one of the most serious public health problems in the case of the rapid aging of the population (7). The aging process can be affected by many external factors, such as exercise and nutrition (8). The effect of EMF exposure on the aging process is also a concern. In 2013, Makarov and Khmelinskii (9) reported that three-dimensional oscillating low-frequency electric EMFs have an effect on the control of the *Drosophila melanogaster* life span, indicating that EMFs might affect the aging process; however, the mechanism is unknown.

Cellular senescence is the origin of aging, which promotes organismal aging and dysfunction (10, 11), and senescence-associated secretory phenotype (SASP) secreted by senescent cells can cause inflammation and change the microenvironment to promote senescence (10). Cellular senescence can be triggered by various stresses, such as activated oncogenes, oxidative stress, shortening of telomeres, DNA damage, and insufficient supplementation (12–14). EMFs have also been reported to induce oxidative stress, DNA damage, and other biological effects, although the results are not definitive (15, 16). Thus, it is necessary to consider the effects of EMFs on cellular senescence.

The effect of EMFs on cellular senescence has been investigated in several studies, but the results have been inconsistent. First, several studies have reported that EMFs can induce cellular senescence. Kim et al. reported that 1.76 GHz radiofrequency EMFs induced cellular senescence in HaCaT human keratinocytes (17). Choi et al. (18) reported that 1.7 GHz radiofrequency EMFs induced senescence in human adipose tissue-derived stem cells (ASCs) and Huh7 liver cancer stem cells. Fathi et al. (19) reported that exposure to 50 Hz MFs induces senescence in rat adipose-derived mesenchymal stem cells. Second, several other studies did not find a significant effect of EMFs on cellular senescence. Alessio et al. (20) reported that 169 MHz radiofrequency EMF exposure had no effect on cellular senescence in human adipose-derived mesenchymal stem cells. Hong et al. (21) reported that exposure to 60 Hz MFs had no effect on cellular senescence in human breast epithelial cells (MCF10A). Finally, some studies have reported that EMFs might be effective in delaying cellular senescence. Perez et al. (22) reported that engineered repeated EMFs therapy upregulates the HSR/HSF1 pathway and delays cellular senescence in young cells. Xu et al. (23) reported that 4 Hz rotating MFs delayed human umbilical vein endothelial cell (HUVEC) senescence. Maredziak et al. (24) reported that static MFs delayed human adipose-derived mesenchymal stem cell senescence. The types of EMFs, cellular models, and endpoints were different in these studies, which might have contributed to the inconsistent results. Although the results were inconsistent, and it was difficult to draw a clear conclusion about the impact of EMFs on cellular senescence, the current available studies provide information that EMFs might have an effect on cellular senescence under specific conditions. However, the specific conditions are unknown, and more information about the effect of EMFs on cell senescence is needed.

In recent years, low-frequency-pulsed (<100 Hz) MF exposure is increasing, that has been frequently applied as a non-invasive, easy, cheap, and reliable alternative method for clinical treatment, such as pain management (25, 26), brain stimulation (27), and bone fracture repair (28). In this study, we investigated the effect of 10 Hz pulsed MF exposure on cellular senescence in 2BS cell line isolated from human fetal lung fibroblasts, a cellular senescence model, and detected cellular senescence-related phenotypes, that is, DNA damage, senescence-associated β-galactosidase (SA-β-gal) activity, SASP, and mitochondrial function. The results showed that intermittent (1 d on/1 d off) exposure to 1.0 mT 10 Hz pulsed MFs for 2 weeks induced DNA damage, but no other significant cellular senescence phenotype in 2BS cells.

## Materials and Methods

### Cell Culture

Human fetal lung fibroblasts (2BS cell line) were obtained from the National Institute of Biological Products (Beijing, China) and have been well-characterized as a cellular senescence model (29–31). Cells were cultured in high-glucose Dulbecco's modified Eagle's medium (DMEM) (Gibco, Grand Island, NY, USA) with 10% fetal bovine serum (FBS, Gibco), 100 U/ml penicillin, and 100 mg/ml streptomycin at 37°C with 5% CO_2_ in an incubator (Thermo Scientific, Shanghai, China). The cells were sub-cultured in a 1:2 or 1:4 ratio when the culture confluence was almost 85%. The formula log_2_(D/D0) was used for the calculation of cell cumulative population doubling (CPD), where D and D0 are the densities of cells at the time of harvesting and seeding, respectively. Cells were considered young at <30 population doubling (PD) and replicative senescent around 55 PD or later.

### The EMF Exposure System

The system was designed and manufactured by CH_HAIL Electronic Devices Inc. (Beijing, China). A pair of Helmholtz coils (CHY15-20J, CH_HAIL), 30 cm in diameter and 9 cm apart, combined with a signal generator (DG1022U, RIGOL, Beijing, China), a power amplifier (CH-EA-500G, CH_HAIL), and a gauss meter (CH-1600, CH_HAIL; Supplementary Figure 1). The system could generate 0–1 kHz, 0–2.0 mT MFs. Helmholtz coils were placed in an incubator (Thermo Scientific) in a humidified atmosphere of 5% CO_2_ at 37°C. Culture flasks and plates were placed between the coils on a transparent polymethylmethacrylate holder, 1 cm below the center of the coils. The coils were oriented on the left and right sides of the flasks and plates. In this configuration, the MFs were parallel to the bottom of the flasks and plates. The temperature of the medium was determined using a medical thermometer, and no significant increase was found compared to the control group during exposure. Total 1.0 mT 10 Hz pulsed MFs (duty ratio = 0.50) were used in the present study. The strengths of the MFs were monitored using a gauss meter at the center of the two coils, and the MF was homogeneous from the coil center to the origin, in the 10 cm of the spherical region, the uniform index <4% (Supplementary Figure 3).

The background static MFs density was 4.32 ± 1.75 μT (Supplementary Figure 2A and Supplementary Data). The root mean square (RMS) value of 10 Hz pulsed MFs density was 1.0 mT (Supplementary Figures 2B,C and Supplementary Data). The pulse period was 100 ms, the pulse width was 50 ms, the rise and fall time was about 20 ns, respectively (Supplementary Figure 2D).

### Exposure Protocol

Cells in the exposure group were exposed to 1.0 mT 10 Hz pulsed MFs (1 d on/1 d off) for 2 weeks. At the same time, cells in the control group were cultured in another incubator (Thermo Scientific) under the same conditions without MF exposure. Cell subcultures progressed during the exposure interval. After exposure, the cells were harvested for further analyses.

### Alkaline Comet Assay

After exposure, the cells were digested, resuspended in a culture medium, and then placed on ice. The sandwich agarose gels were made up of 0.65% normal-melting agarose that had been pre-coated on a slice and 0.65% low-melting agarose mixed with cells on normal-melting agarose. The cells were lysed in lysis buffer containing 1% Triton X-100 at 4°C for 1 h and then enzymatically hydrolyzed in lysis buffer containing 0.5 mg/ml DNase-free proteinase K (Beyotime, Shanghai, China) at 37°C for 2 h. Before electrophoresis, DNA in cells was unwind in ice-cold alkaline electrophoresis solution for 20 min and then electrophoresis at 20 V for 20 min. After electrophoresis, cells were neutralized in Tris buffer (0.4 M, pH 7.5) for 2 × 5 min. DNA “comets” were stained by Gel-red (Beyotime) and photographed by a fluorescence microscope (Zeiss, Oberkochen, German). Approximately 30 comets for each sample were calculated using the CASP 1.2.2 software (Krzysztof Konca, Wroclaw, Poland). Additional details can be found in a previously described protocol (32).

### Western Blotting

After exposure, cells were washed with ice-cold PBS and then lysed with radioimmunoprecipitation assay (RIPA) lysis buffer (P0013B, Beyotime) with 1 × protease inhibitor cocktail (Roche Diagnostics, Indianapolis, IN, USA) on ice. Cell lysates were separated by electrophoresis on 10% sodium dodecyl sulfate (SDS)-polyacrylamide gels, transferred to polyvinylidene fluoride (PVDF) membranes (Bio-Rad Laboratories, Hercules, CA, USA), blocked with 5% skimmed milk PBS with 0.05% Tween 20, and blotted with the primary antibody for 2 h at room temperature. This was followed by incubation with horseradish peroxidase (HPR)-conjugated goat anti-mouse or goat anti-rabbit IgG at room temperature for 1 h. Immunoreactive bands were detected using the enhanced chemiluminescence (ECL) method. The primary rabbit anti-γH2AX antibody was purchased from Beyotime Technology and anti-p21^Waf1^, p16^INK4a^, and GAPDH antibodies were from Cell Signaling Technology (Danvers, MA, USA), and the anti-p53 antibody was from Santa Cruz Biotechnology (Santa Cruz, CA, USA).

### SA-β-Gal Staining

Senescence-associated β-galactosidase staining was performed using a commercial kit (C0602, Beyotime). In brief, after exposure, the cells were fixed for 15 min and then incubated with staining solution at 37°C without a CO_2_ supply overnight. The cells were visualized using a microscope (Zeiss), and the percentage of positively stained cells was calculated. A total of 200 cells per sample were analyzed.

### Quantitative Real-Time PCR

Total RNA was extracted from the cells using a total RNA extraction reagent (TRIzol, Thermo Scientific) and transcribed to cDNA using a reverse transcription kit (BL699A, Biosharp, Hefei, China) with a PCR system (Bio-Rad). RNA expression was quantified using an SYBR Green qPCR Mix kit (BL698A, Biosharp) using a qPCR system (Roche, Basel, Switzerland). The expression of miRNA was defined from the threshold cycle, and relative expression levels were calculated using the 2^−Δ*ΔCt*^ method after normalization with reference to the expression of 18S mRNA. Primer sequences were obtained from a published article (33) and were synthesized by Tsingke Biotechnology Co., Ltd. (Hangzhou, China).

### Cell Cycle Analysis

The cells were harvested, resuspended in pre-cooled PBS with 70% ethanol, and stored at −20°C overnight. Cells were then stained with 50 μg/ml propidium iodide (Beyotime) diluted in PBS containing 50 mg/ml RNase A (Thermo Scientific) at room temperature (25°C) for 30 min. The cell cycle distribution was analyzed by flow cytometry (Cytoflex, Beckman Coulter, CA, USA), in which 1 × 10^4^ events per sample were acquired, and the proportions of cells in the G0/G1, S, and G2/M phases were determined.

### Cell Viability Analysis

After exposure, the cells were detached using 200 μl of 0.25% trypsin-EDTA (Gibco) and resuspended in 500 μL of culture medium. Then, 3,000 cells per well were seeded into a 96-well-plate (Corning, NY, USA). Cell viability was determined using Cell Counting Kit-8 (CCK-8, Dojindo Molecular Technologies, Kumamoto, Japan) at 24 and 48 h after seeding. The values were detected using a microplate reader (Tecan, Hombrechtikon, Switzerland).

### Cell Proliferation Assay

Cell proliferation was determined using a 5-ethyny-2′-deoxyuridine (EdU) cell proliferation kit (C0071S, Beyotime). In brief, cells were incubated with EdU for 1 h and then harvested. Cells were fixed in 70% ethanol at −20°C overnight. EdU signal per cell was detected by flow cytometry (Cytoflex, Beckman Coulter). More details can be found in the instructions of manufacturer.

### Intracellular Reactive Oxygen Species Measurement

Intracellular ROS were detected using 2,7-dichlorodihydrofluorescein diacetate (H_2_DCFDA) (Beyotime). After exposure, cells were washed twice with an FBS-free culture medium and then incubated with 10 μM H_2_DCFDA diluted in an FBS-free culture medium at 37°C for 20 min. The cells were then harvested and washed with PBS. H_2_DCFDA signaling was determined using flow cytometry (Cytoflex, Beckman Coulter).

### Mitochondrial Membrane Potential Determination

Mitochondrial membrane potential in cells was determined using an MMP assay kit with JC-1 (Beyotime). Briefly, cells were detached and resuspended in a 0.5 ml culture medium. The cells were then incubated with JC-1 at 37°C for 20 min, and then the intensities of JC-1 (red and green fluorescence) were determined by flow cytometry (Cytoflex, Beckman Coulter).

### Adenosine 5′-Triphosphate (ATP) Detection

Adenosine 5′-triphosphate levels were determined using an ATP Assay Kit (Beyotime). Briefly, after exposure, the cells were lysed at 4°C. After centrifugation, ATP and protein concentrations in the supernatant were determined. The concentration of ATP per unit protein in each sample was then calculated. A microplate reader (Tecan) was used to read the data.

### Enzyme-Linked Immunosorbent Assay

The levels of interleukin (IL)-6, CXC chemokine ligand 8 (IL-8), and growth-regulated oncogene alfa (GRO-α) in the culture medium were determined using commercial ELISA kits (SolelyBio, Shanghai, China). Briefly, after exposure, the cell culture medium was collected and added to the test plate. After reaction at 37°C for 30 min, the culture medium was removed, and an enzyme labeling reagent was added to the plate. After reaction at 37°C for 30 min, the enzyme labeling reagent was removed, and a chromogenic solution was added to the plate. After reaction at 37°C for 10 min, the reaction stop solution was added to stop the reaction, and the values were immediately detected using a microplate reader (Tecan). More details can be found in the manufacturer's instructions.

### Statistical Analysis

All experiments were performed in triplicate at least. Student's *t*-test was applied for comparisons between two groups in which the data followed a normal distribution, and Wilcoxon rank-sum test was applied when the data were not a normal distribution, all the data were analyzed in R software (Version 3.6.1), and the differences were considered statistically significant at *P* < 0.05.

## Results

### Effect of 10 Hz Pulsed MFs on DNA Damage in 2BS Cells

**Figure 1:**
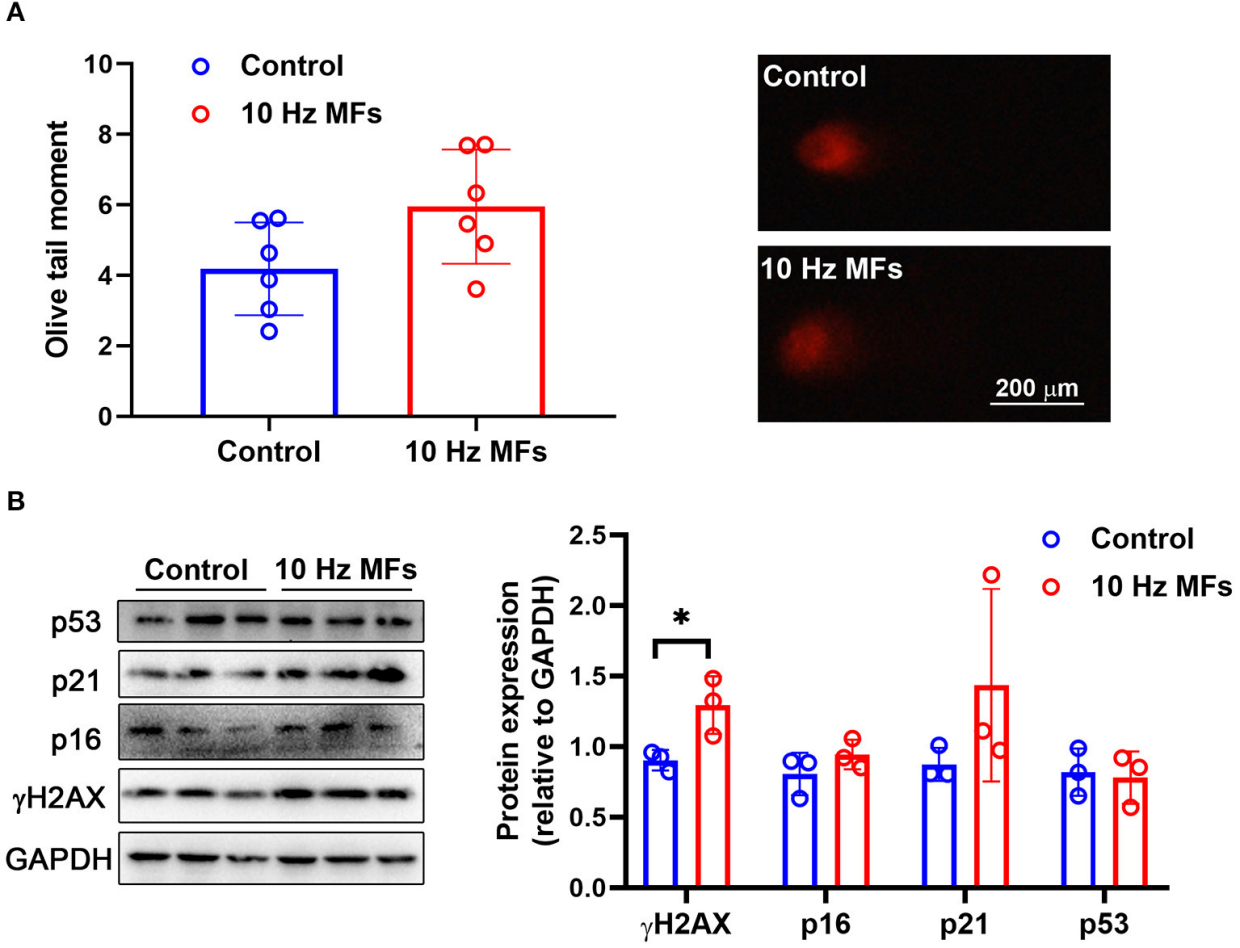
The effect of 10 Hz pulsed MFs exposure on DNA damage and p16, p21, and p53 expressions in 2BS cells. **(A)** The statistical values of DNA fragmentation (Olive tail moment) in 2BS cells of control and 10 Hz pulsed MFs exposure group and representative images of alkaline comet assay; **(B)** the statistical graphic of protein expression (relative to GAPDH) and blot images of γH2AX, p16, p21, and p53 in 2BS cells. All data were presented as mean ± SD. *P < 0.05.

DNA damage accumulation is not only a phenotype but also a cause of cellular senescence (11, 34). In our results, the alkaline comet assay showed that exposure to 1.0 mT 10 Hz pulsed MFs for 2 weeks increased DNA fragmentation, but the difference was not statistically significant (Figure 1A). To further confirm this, we detected γH2AX, a sensitive marker of DNA damage (35), and found that 10 Hz pulsed MFs exposure significantly increased γH2AX in 2BS cells compared to the control group (Figure 1B), indicating that 10 Hz pulsed MFs exposure has an effect on DNA damage in 2BS cells.

### Effect of 10 Hz Pulsed MFs on p16, p21, and p53 Expression in 2BS Cells

Cellular senescence is characterized as a stable and terminal state of growth arrest in which cells are unable to proliferate (36). Accumulated DNA damage activated DNA damage response (DDR), a factor that activates cell cycle arrest. At the bottom of the DDR cascade, the tumor suppressor p53, the cyclin-dependent kinase inhibitor p21, and CDK4 and CDK6 inhibitor p16 all play critical roles in senescence entry and the maintenance of the senescence phenotype (36). We determined the expressions of p16, p21, and p53 *via* western blot assay, and the results showed that 10 Hz pulsed MF exposure did not significantly increase p16, p21, or p53 expression levels in 2BS cells compared to the control group (Figure 1B).

### Effect of 10 Hz Pulsed MFs on SA-β-gal Staining in 2BS Cells

**Figure 2:**
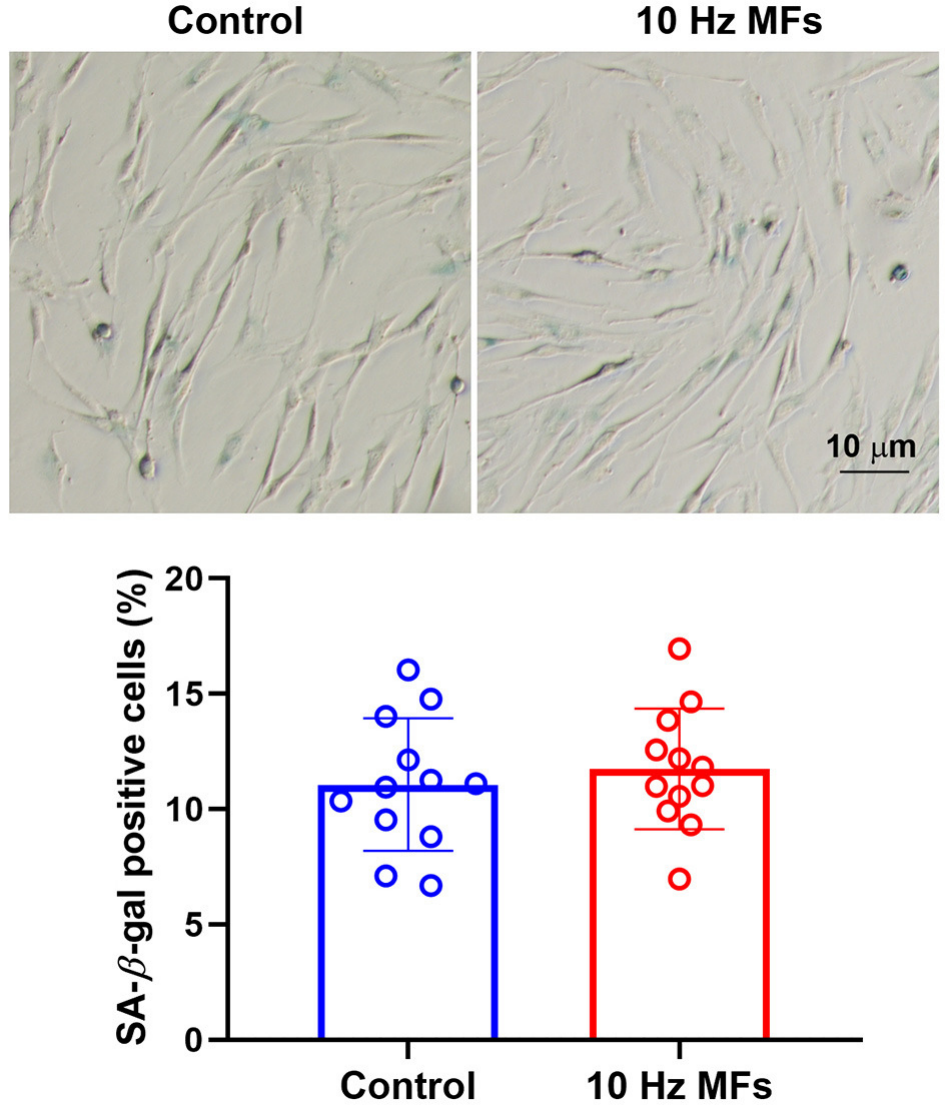
The effect of 10 Hz pulsed MFs exposure on SA-β-gal staining in 2BS cells. Representative images show the SA-β-gal staining in 2BS cells of control and 10 Hz pulsed MFs exposure group, bar = 10 μm, and the graphic shows the statistical values of SA-β-gal staining (Percentage of positive staining cells), data were presented as mean ± SD. SA-β-gal, senescence-associated β-galactosidase.

Senescence-associated β-galactosidase is a lysosomal enzyme whose accumulation is the most widely used biomarker for detecting cellular senescence (37). The results of the SA-β-gal staining assay showed that 10 Hz pulsed MF exposure did not significantly increase the percentage of positively stained cells in 2BS cells compared to the control group (Figure 2).

### Effect of 10 Hz Pulsed MFs on SASP in 2BS Cell

**Figure 3:**
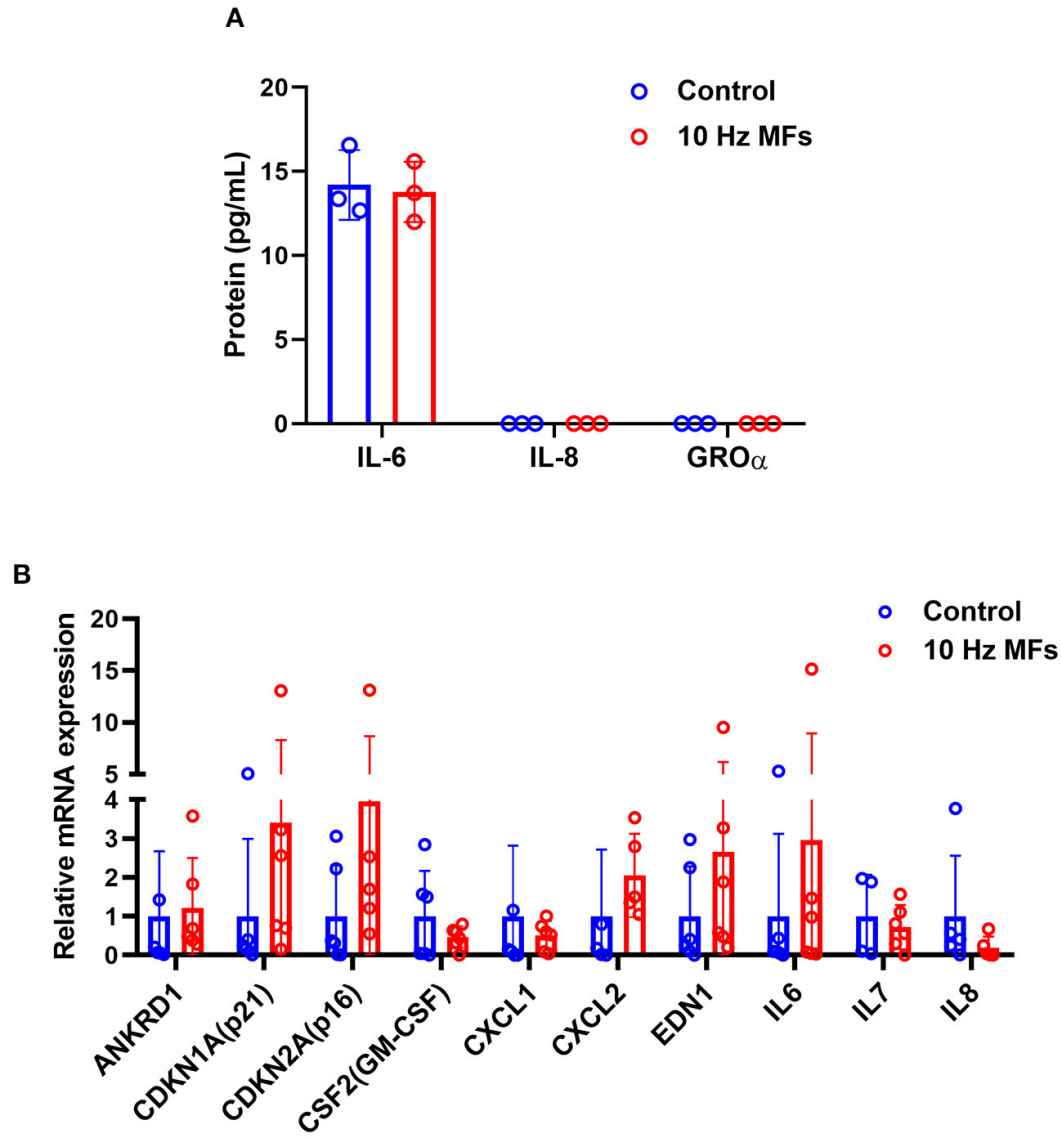
The effect of 10 Hz pulsed MFs exposure on SASP in 2BS cells. **(A)** The levels of IL-6, IL-8, and GROα in the culture medium and **(B)** mRNA expression of ANKRD1, CDKN1A, CDKN2A, CSF2, CXCL1, CXCL2, EDN1, IL-6, IL-7, and IL-8 in 2BS cells of control and 10 Hz pulsed MFs exposure group. Data were presented as mean ± SD. SASP, senescence-associated secretory phenotype. IL-8, CXC chemokine ligand 8; IL-6, interleukin 6; GRO-α, growth-regulated oncogene alfa.

Senescence-associated secretory phenotype is a typical phenotype of senescent cells, such as pro-inflammatory cytokines, chemokines, growth factors, and proteases (37). SASP is also a potential mechanism through which senescent cells exert their pleiotropic biological functions (37). The levels of proinflammatory IL-6, CXC chemokine ligand 8 (IL-8), and growth-regulated oncogene (GRO-α) in the culture medium of 2BS cells were determined by ELISA. The results showed that 10 Hz pulsed MF exposure did not increase IL-6 secretion compared to the control group, and IL-8 and GRO-α levels were below the detection limit (Figure 3A). The mRNA expression of several SASP factors was determined by qPCR. The results showed that ANKRD1, CDKN1A, CDKN2A, CSF2, CXCL1, CXCL2, EDN1, IL-6, IL-7, and IL-8 were not significantly affected by 10 Hz pulsed MF exposure compared to that in the control group (Figure 3B).

### Effect of 10 Hz Pulsed MFs on Cell Proliferation and Cell Cycle Progression in 2BS Cells

**Figure 4:**
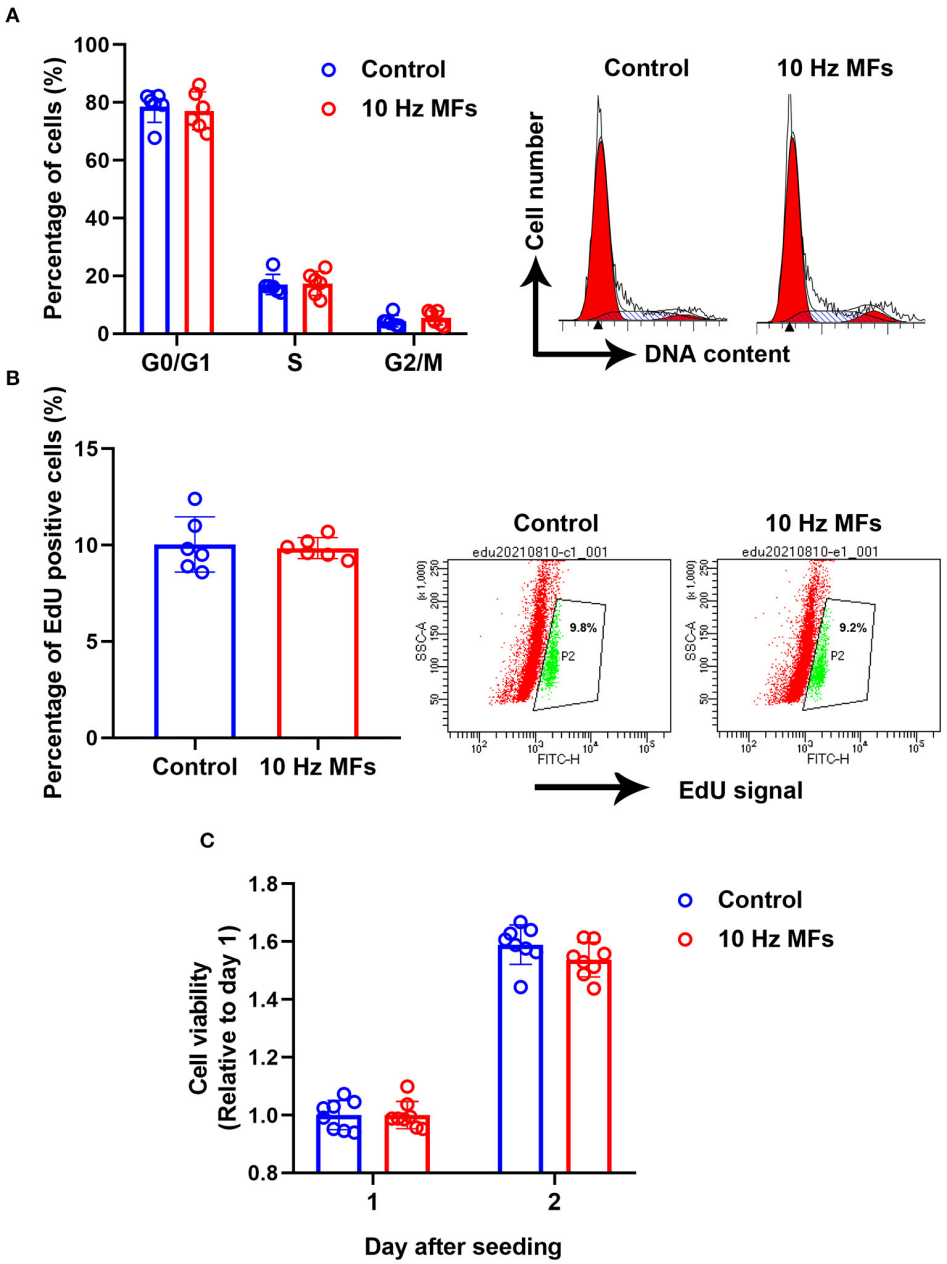
The effect of 10 Hz pulsed MFs exposure on cell cycle progression and proliferation. **(A)** Cell cycle distributions were determined by PI staining, **(B)** cell proliferation was determined by EdU assay, and **(C)** cell viability was determined by CCK8 assay of 2BS cells in control and 10 Hz pulsed MFs exposure group. Data were presented as mean ± SD. MFs, magnetic fields.

Cellular senescence is defined as the irreversible arrest of cell proliferation in response to exogenous or endogenous stimuli (38). Thus, we determined cell proliferation and cell cycle progression. The results showed that neither cell proliferation nor cell cycle progression was significantly affected in 10 Hz pulsed MFs exposed 2BS cells compared to that in the control group (Figure 4).

### Effect of 10 Hz Pulsed MFs on ROS Generation, MMP, and ATP Levels in 2BS Cells

**Figure 5:**
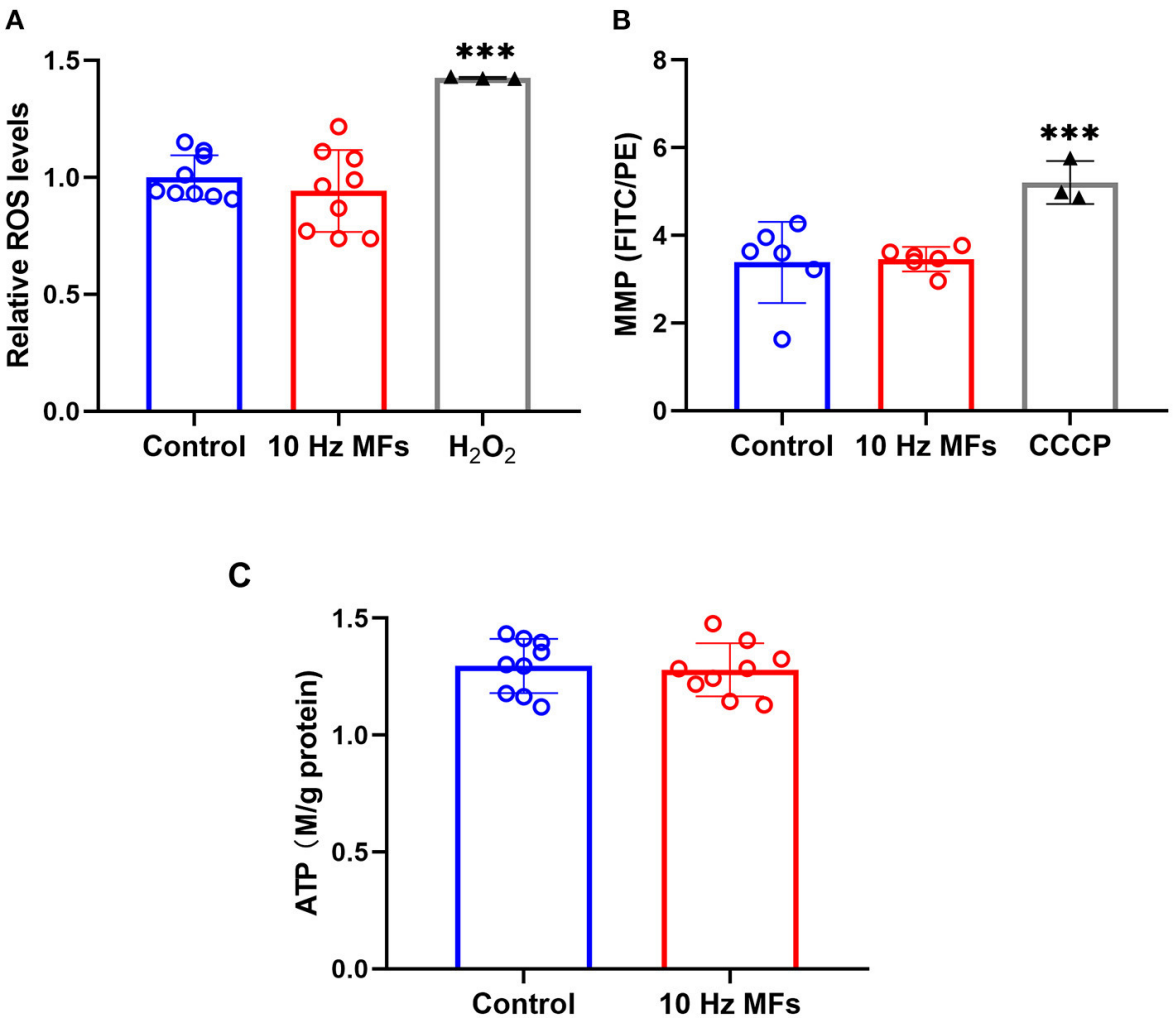
The effect of 10 Hz pulsed MFs exposure on ROS, MMP, and ATP levels in 2BS cells. **(A)** Relative intracellular ROS levels were determined by H_2_DCFDA, **(B)** MMP determined by JC-1, and **(C)** ATP concentration in 2BS cells of control and 10 Hz pulsed MFs exposure group. Hydrogen peroxide (H_2_O_2_) and CCCP were applied as the positive control, respectively. Data were presented as mean ± SD. ***P < 0.001. MFs, magnetic fields; ROS, reactive oxygen species; H_2_DCFDA, 2,7-dichlorodihydrofluorescein diacetate; MMP, Mitochondrial membrane potential; ATP, adenosine 5′-triphosphate.

Mitochondria are important organelles that provide energy for cell metabolism. Mitochondrial dysfunction is a hallmark of cellular senescence (11), which leads to insufficient energy supply and excessive ROS generation, such as cell damage and aging (38, 39). Thus, we determined the cellular ROS, MMP, and ATP levels. The results showed that neither ROS levels, MMP, nor ATPs were significantly changed in 2BS cells that were exposed to 10 Hz pulsed MFs compared to that in the control group (Figure 5).

## Discussion

As the largest risk factor for a multitude of age-related diseases, rapid population aging has led to a global burden of late-life disease (7, 40). Aging-related senescence leads to progressive deterioration of bodily functions, which is associated with a loss of complexity in a wide range of physiological processes and anatomic structures (40). The aging process is not only the result of genotype but also closely related to external factors. For decades, the rapid increase in the use of EMFs has raised public concerns about the potential biological effects of EMFs, and the effect of EMFs on aging has also attracted attention. Cellular senescence is the basis of aging, which results in tissue and organ senescence and leads to body dysfunction (10, 11). Thus, it is important to understand the effects of EMF exposure on cellular senescence. Although there have been several investigations, the results were inconsistent and the effects of EMFs on cellular senescence are not completely understood, therefore, more studies are required. In this study, we investigated the effect of 10 Hz pulsed MFs on cellular senescence in human fetal lung fibroblasts (2BS) by detecting several hallmarks of senescent cells, such as DNA damage, p53, p21, and p16 expression levels, SA-β-gal activity, SASP, and mitochondria function (33) and found that 10 Hz pulsed MFs only increased DNA damage but no other senescent phenotypes in 2BS cells.

Impaired DNA damage is not only a hallmark of aging but also a causal factor of the aging process, which will constantly activate DDR to arrest the cell cycle and growth (11, 34). In this study, the results of the alkaline comet assay and γH2AX expression levels showed that 10 Hz pulsed MF exposure induced DNA damage in 2BS cells (Figure 1), but no other significant senescence phenotypes were observed compared to the control, such as p53, p21, and p16 (Figure 1), which are important factors in DNA damage-induced cellular senescence (34, 36), and cell cycle progression and proliferation (Figure 4) were also not significantly changed. These results suggest that the DNA damage induced by 10 Hz pulsed MFs is repairable or too small to induce significant senescence in 2BS cells after 2 weeks of exposure.

To further investigate the effect of 10 Hz pulsed MFs on cellular senescence, SA-β-gal activation, and SASP factors, two typical characteristics of senescent cells were determined in this study. SA-β-gal staining is a common and widely used marker for detecting cell senescence (41). SASP is comprised of a packet of pro-inflammatory cytokines, chemokines, growth factors, and proteases that are important for senescent cells to exert their pleiotropic biological functions, such as self-reinforcing senescence or affecting the microenvironment (36). In this study, no significant changes in SA-β-gal activation (Figure 2) and several SASP levels (Figure 3) were detected in the 10 Hz pulsed MF exposure group compared to that in the control group. These results suggested that 10 Hz pulsed MFs do not induce significant cell senescence in 2BS cells.

Senescent cells are also characterized by mitochondrial dysfunction, which plays an important role in the establishment of senescence, such as increased oxidative stress (36). In this study, to determine the function of mitochondria, we detected MMP, ROS, and ATP generation, and no significant effect was observed (Figure 5), indicating that mitochondrial function was not affected by 10 Hz pulsed MF exposure in 2BS cells.

The 2BS cells used in this study were isolated from human fetal lung fibroblasts by the National Institute of Biological Products (Beijing, China) and have been well-characterized and widely used as a cellular senescence model (29–31). Cells are defined as young at a number lower than 30 PD and replicative senescent around 55 PD or later (39). In this study, exposure to MFs was started at 30 PD and ended at 40–42 PD of the 2BS cells that the cells were in the process of aging but not completely senescent. Thus, this study only investigated the effect of 10 Hz pulsed MFs on the aging process of 2BS cells, whether 10 Hz pulsed MF exposure has an effect on young or senescent 2BS cells requires further study.

In conclusion, under current conditions, intermittent (1 d on/1 d off) exposure to 10 Hz pulsed MFs at 1.0 mT for 2 weeks did not induce significant cellular senescence in 2BS cells.

## Data Availability Statement

The raw data supporting the conclusions of this article will be made available by the authors, without undue reservation.

## Author Contributions

CS contributed to the idea, design, experiments, data analysis, and manuscript writing. ZH did the comet assay, ELISA, qPCR, and western blot. HQ did the flow cytometry and data analysis. JZ, SW, XX, and SY contributed to the data analysis and manuscript writing. GM contributed to the idea, design, and manuscript writing. All authors agree to be accountable for the content of the work.

## Funding

This work was supported by grants from the Medical Science and Technology Project of Zhejiang Province (2021KY005, 2020KY387, and 2019KY257), National Science Foundation of China (31700734, 81973011, and 81701393), Zhejiang Provincial Natural Science Foundation (LGF21H250002), and Chinese Traditional Medicine Science and Technology Projects of Zhejiang Province (2021ZB002).

## Conflict of Interest

The authors declare that the research was conducted in the absence of any commercial or financial relationships that could be construed as a potential conflict of interest.

## Publisher's Note

All claims expressed in this article are solely those of the authors and do not necessarily represent those of their affiliated organizations, or those of the publisher, the editors and the reviewers. Any product that may be evaluated in this article, or claim that may be made by its manufacturer, is not guaranteed or endorsed by the publisher.
