# Supplementary material for: Exposure to 10 Hz Pulsed Magnetic Fields Do Not Induce Cellular Senescence in Human Fetal Lung Fibroblasts
Source: Front Public Health. 2021 Nov 11;9:761069. doi: 10.3389/fpubh.2021.761069 (PMC8632261; doi:10.3389/fpubh.2021.761069)
Supplement: Supplementary file 1 [file Data_Sheet_1.docx]

Supplementary Material

**Supplementary Figures**


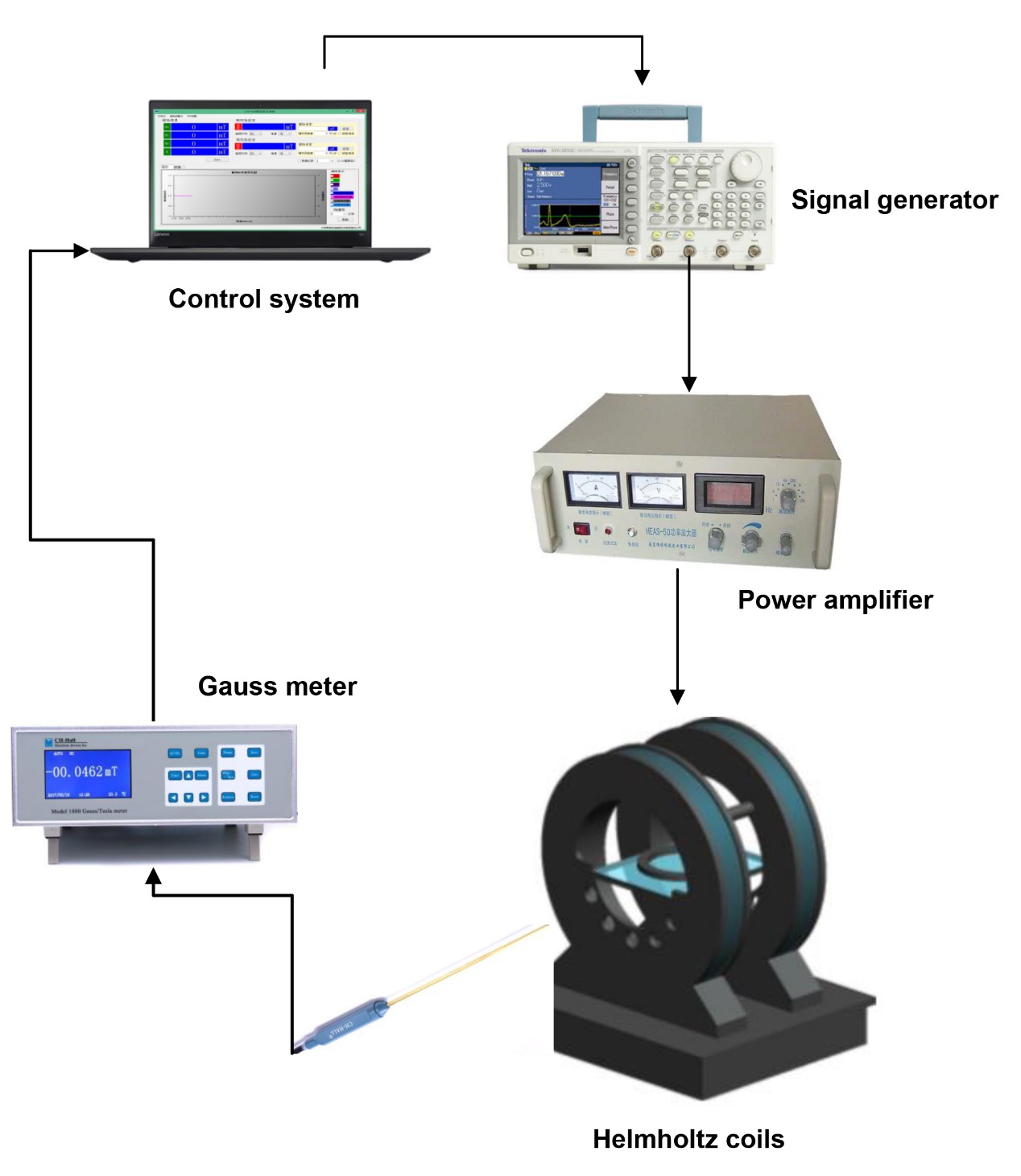


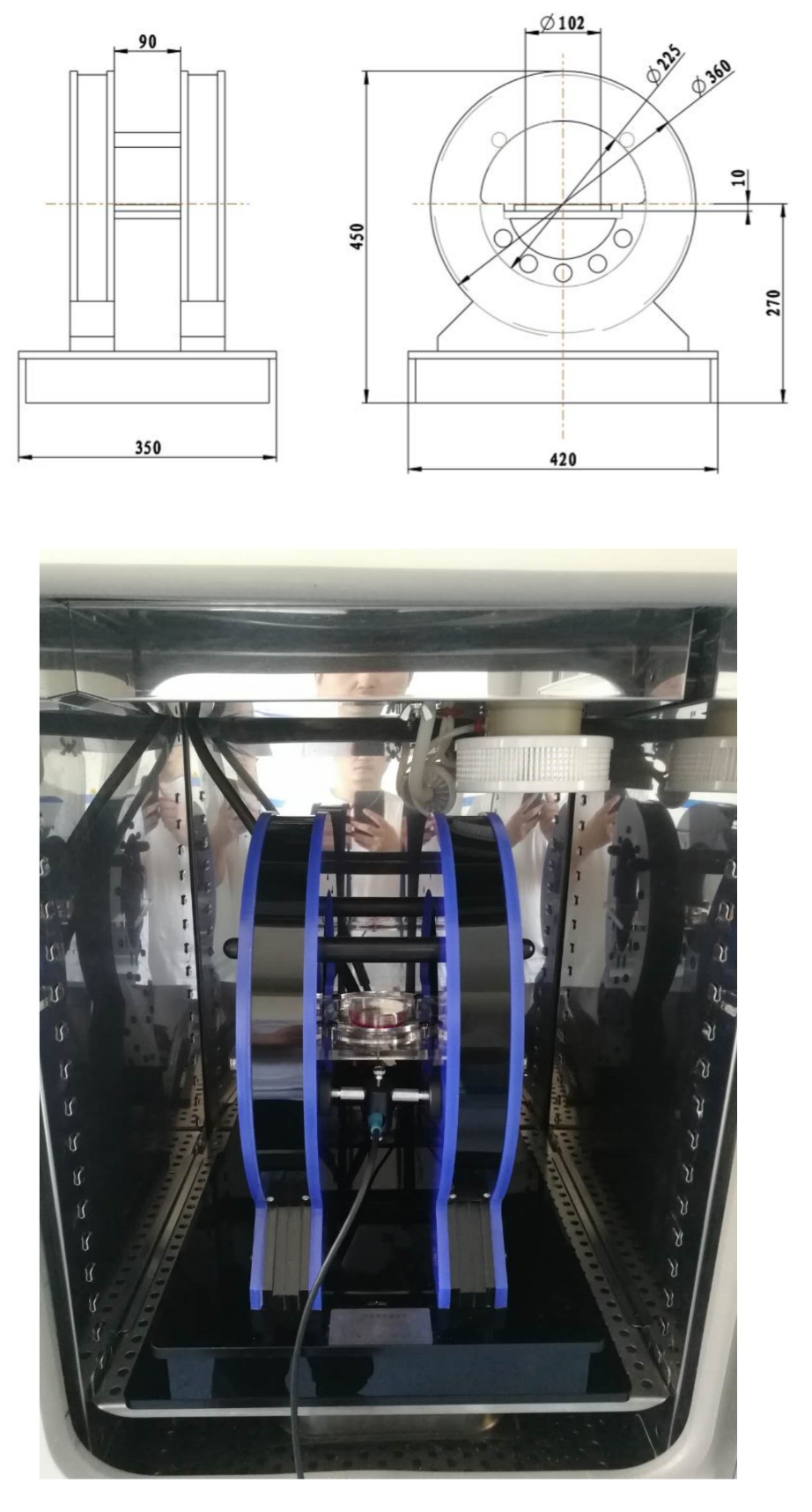


**Supplementary Figure 1**. The components of the electromagnetic fields generation system and the structure and size of the pair Helmholtz coils.


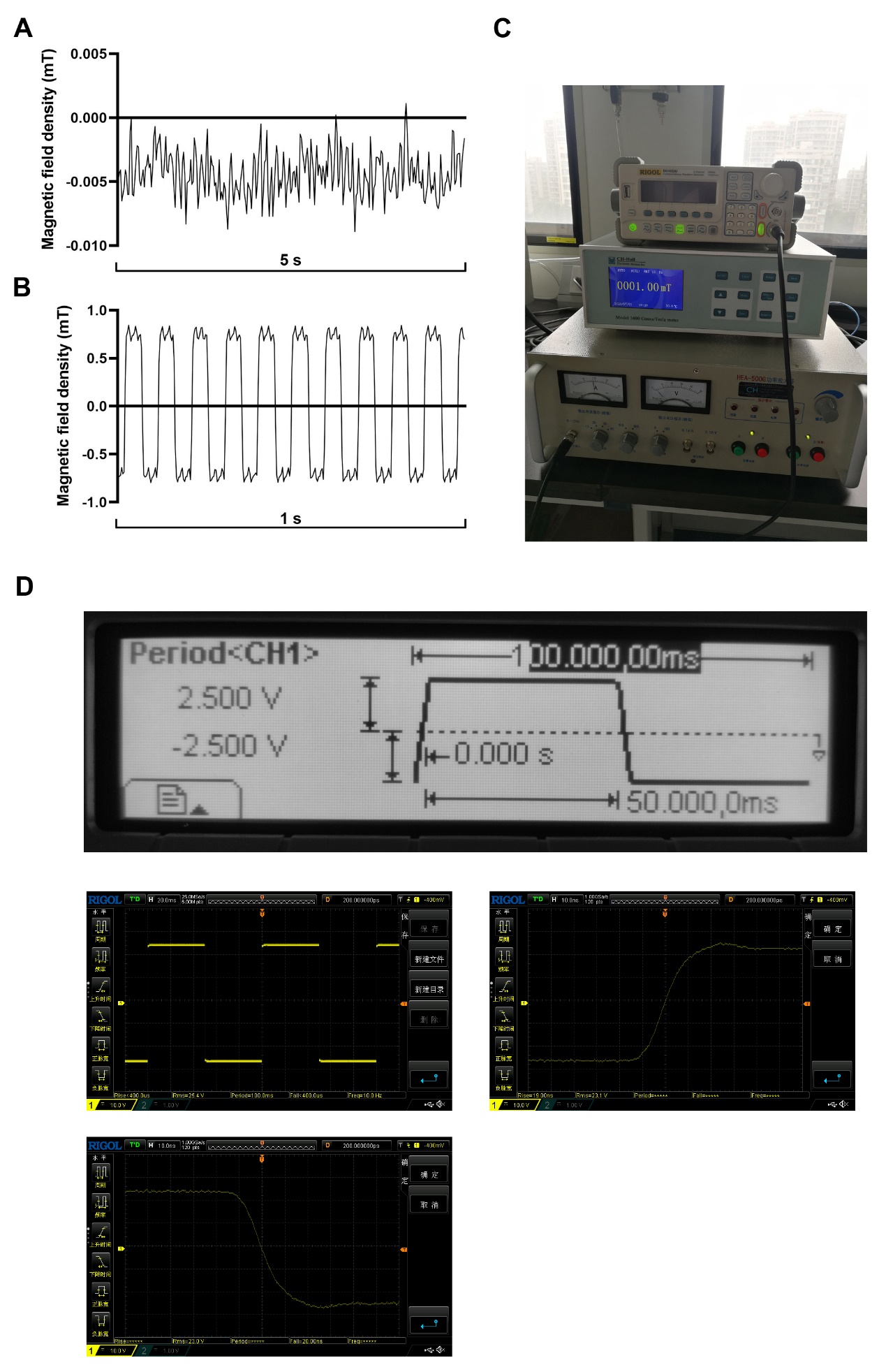


**Supplementary Figure 2.** The density and wave form of magnetic fields. (A) Background levels of static magnetic fields; (B) The density of magnetic fields during exposure; (C) The value (RMS) of pulsed magnetic fields detected by Gauss meter during exposure; (D) The wave form of the 10 Hz pulsed MFs signal.


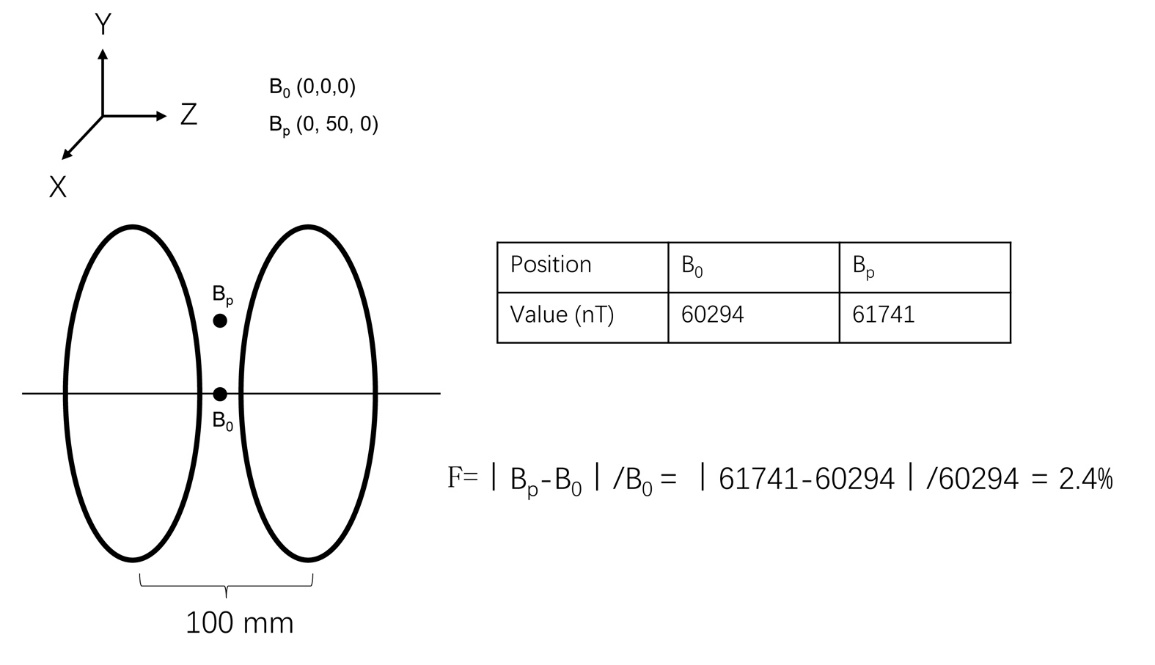


**Supplementary Figure 3.** The density of magnetic fields of the helmholtz coils were measured under direct current (1 A) at B_0_ (the center of the coils) and B_p_ (the edge of the 10 cm spherical region). The calculated uniform index (F) was 2.4%.


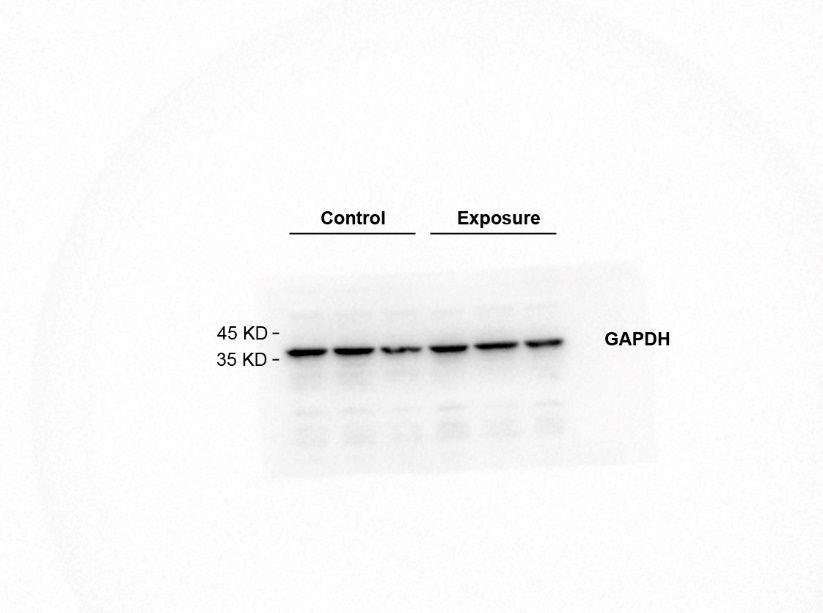

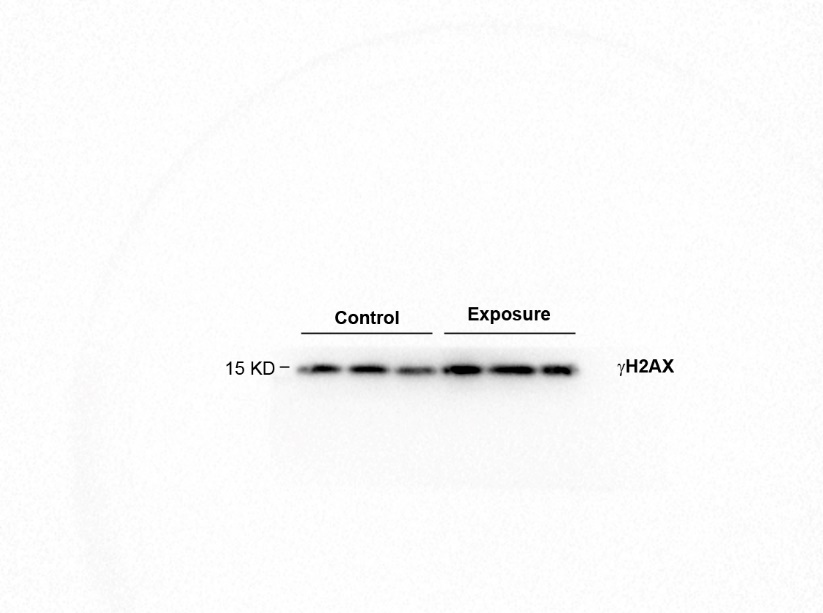

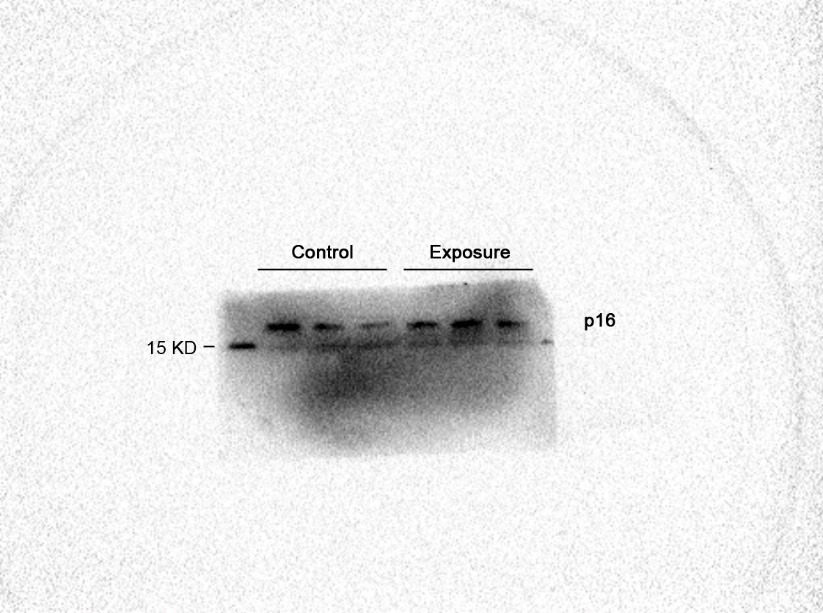

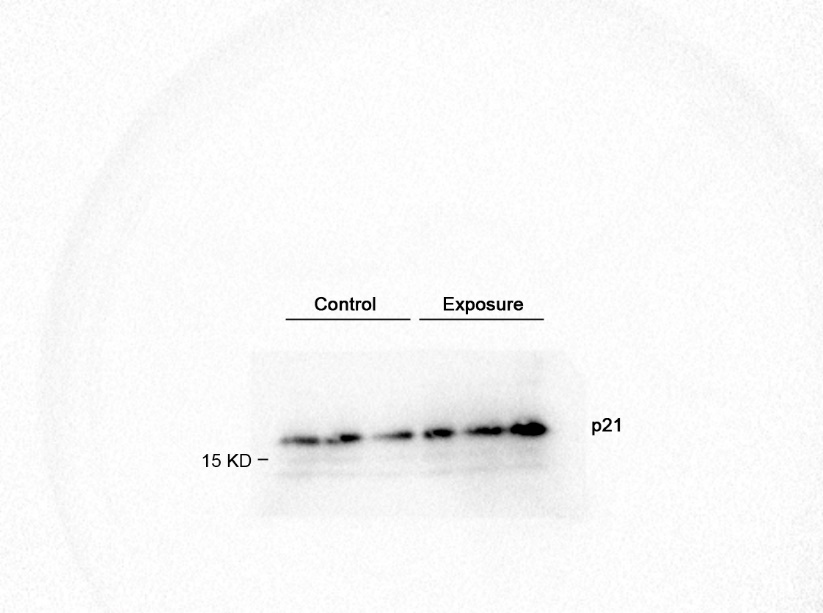

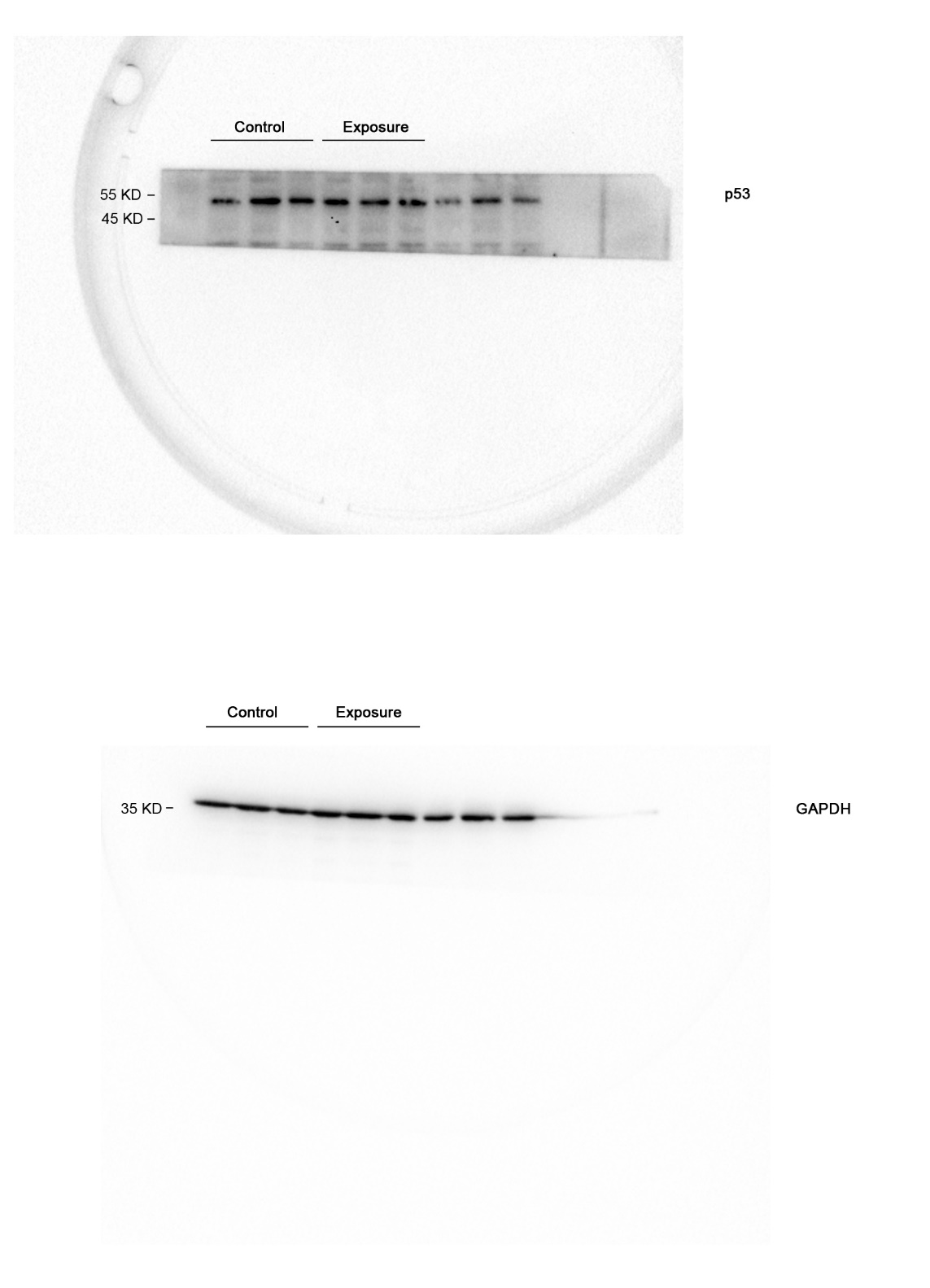


**Supplementary Figure 4.** Raw scans of gels of western blot.

**Supplementary Tables**

**Supplementary Table 1.** The sequence of primers

| **Human Primers** | **Forward** | **Reverse** |
| --- | --- | --- |
| *18S* | CCCTATCAACTTTCGATGGTAGTCG | CCAATGGATCCTCGTTAAAGGATTT |
| *ANKRD1* | AGT AGA GGA ACT GGT CACTGG | TGG GCT AGA AGT GTCTTC AGA T |
| *CDKN1A (p21)* | GAC ACCACT GGA GGG TGA CT | CAGGTC CAC ATG GTC TTC CT |
| *CDKN2A (p16)* | CCAACGCACCGAATAGTTACG | GCGCTGCCCATCATCATG |
| *CSF2 (GM-CSF)* | GGCCCCTTGACCATGATG | TCTGGGTTGCACAGGAAGTTT |
| *CXCL1* | GAAAGCTTGCCTCAATCCTG | CACCAGTGAGCTTCCTCCTC |
| *CXCL2* | AACTGCGCTGCCAGTGCT | CCCATTCTTGAGTGTGGCTA |
| *EDN1* | CAG CAGTCT TAG GCG CTG AG | ACTCTT TAT CCA TCA GGG ACG AG |
| *IL6* | CCGGGAACGAAAGAGAAGCT | GCGCTTGTGGAGAAGGAGTT |
| *IL7* | CTCCAGTTGCGGTCATCATG | GAGGAAGTCCAAAGATATACCTAAAAGAA |
| *IL8* | CTTTCCACCCCAAATTTATCAAAG | CAGACAGAGCTCTCTTCCATCAGA |
